# Nevertheless, She Resisted – Role of the Environment on *Listeria monocytogenes* Sensitivity to Nisin Treatment in a Laboratory Cheese Model

**DOI:** 10.3389/fmicb.2020.00635

**Published:** 2020-04-09

**Authors:** L. O. Henderson, B. J. Erazo Flores, J. Skeens, D. Kent, S. I. Murphy, M. Wiedmann, V. Guariglia-Oropeza

**Affiliations:** ^1^Department of Food Science, Cornell University, Ithaca, NY, United States; ^2^Universidad de Puerto Rico, Mayagüez, Puerto Rico

**Keywords:** *Listeria monocytogenes*, cell wall, *dltA*, *mprF*, nisin

## Abstract

The growth of *Listeria monocytogenes* on refrigerated, ready-to-eat food products is a major health and economic concern. The natural antimicrobial nisin targets the bacterial cell wall and can be used to inhibit *L. monocytogenes* growth on cheese. Cell wall composition and structure, and therefore the efficacy of cell wall acting control strategies, can be severely affected by environmental and stress conditions. The goal of this study was to determine the effect of a range of pH and temperatures on the efficacy of nisin against several strains of *L. monocytogenes* in a lab-scale, cheese model. Cheese was made with or without the addition of nisin at different pH and then inoculated with *L. monocytogenes*; *L. monocytogenes* numbers were quantified after 1, 7, and 14 days of incubation at 6, 14, or 22°C. While our data show that nisin treatment is able to reduce *L. monocytogenes* numbers, at least initially, growth of this pathogen can occur even in the presence of nisin, especially when cheese is stored at higher temperatures. Several environmental factors were found to affect nisin efficacy against *L. monocytogenes*. For example, nisin is more effective when cheese is stored at lower temperatures. Nisin is also more effective when cheese is made at higher pH (6 and 6.5), compared to cheese made at pH 5.5, and this effect is at least partially due to the activity of cell envelope modification genes *dltA* and *mprF*. Serotype was also found to affect nisin efficacy against *L. monocytogenes*; serotype 4b strains showed lower susceptibility to nisin treatment compared to serotype 1/2 strains. Overall, our results highlight the importance of considering environmental conditions specific to a food matrix when developing and applying nisin-based intervention strategies against *L. monocytogenes*.

## Introduction

Controlling *Listeria monocytogenes* in food is essential to food safety due to the high mortality rate associated with listeriosis, especially among susceptible populations, such as pregnant women, newborns, the elderly, and those with compromised immune systems (Farber and Peterkin, 1991; Jackson et al., 2018). *L. monocytogenes* can also cause disease in animals and is found in both natural and food-processing environments (Nightingale et al., 2004). *L. monocytogenes* can be classified into different serotypes, which are associated with specific environments and differ in their ability to cause disease (Orsi et al., 2011). Each serotype belongs to at least one of four lineages: I, II, III, and IV (Piffaretti et al., 1989; Rasmussen et al., 1995; Wiedmann et al., 1997; Roberts et al., 2006; Ward et al., 2008); however, the majority of *L. monocytogenes* isolates commonly associated with human clinical cases belong to lineages I (serotype 1/2b and 4b) (Orsi et al., 2011), whereas lineage II (serotypes 1/2a) isolates are more prevalent in food products and natural and farm environments (Kabuki et al., 2004; Manfreda et al., 2005).

*L. monocytogenes* is of particular concern in ready-to-eat (RTE) dairy foods that allow *L. monocytogenes* growth, such as Hispanic-style fresh cheese (Jackson et al., 2018). High water activity, low salt content, and near neutral pH make Hispanic-style fresh cheese an ideal environment for survival and growth of a number of foodborne pathogens (Leggett et al., 2012), including *L. monocytogenes* (Uhlich et al., 2006; Ibarra-Sánchez et al., 2017). Furthermore, *L. monocytogenes* can tolerate a number of environmental stressors associated with foods, including a wide range of temperatures (0–45°C), pH (4.4–9.4), and high salt concentrations (13–14 w/v%) (Swaminathan et al., 2007). The ability of *L. monocytogenes* to survive adverse environmental conditions increases the likelihood of transmission from the environment to humans via contaminated food products. Critical points for controlling *L. monocytogenes* in the food supply are prevention of post-processing contamination and/or reformulation of RTE foods using antimicrobials (Ilsi Research Foundation/Risk Science Institute and Expert Panel on Listeria monocytogenes in Foods, 2005).

One control strategy currently used for *L. monocytogenes* on RTE foods is the addition of bacteriocins. Bacteriocins are ribosomally synthesized cationic antimicrobial peptides (CAMPs) produced by bacteria that inhibit the growth of a broad spectrum of pathogens (Heng et al., 2007). Some bacteria that produce bacteriocins are considered food grade by the United States Food and Drug Administration (FDA) (Chen and Hoover, 2006), making them useful for food preservation (Hu et al., 2015). Although, there are a number of bacteriocins that have been studied for their antimicrobial properties, nisin and pediocin are the only commercially available, FDA-approved bacteriocins used in a variety of food products (Chen and Hoover, 2006; Garsa et al., 2014). Of these two bacteriocins, nisin is the most widely used (Ross et al., 2002). Nisin is produced by *Lactococcus lactis* and is active against a broad number of Gram-positive bacteria (O’Connor et al., 2015). Nisin has two modes of action: (i) binding to lipid II, a precursor molecule in cell-wall synthesis, thus preventing synthesis of the cell-wall component peptidoglycan (Wiedemann et al., 2001; Hasper et al., 2006) and (ii) aggregating in complexes that create pores in the bacterial cell membrane, subsequently causing cell lysis (Brotz et al., 1998; Hasper et al., 2006). Previous studies have shown that nisin can inhibit growth of *L. monocytogenes* on cheese (Van Tassell et al., 2015), hotdogs (Vijayakumar and Muriana, 2017), and smoked meats (Nilsson et al., 1997), suggesting that the use of nisin or other bacteriocins can reduce pathogen prevalence and/or levels and may consequently reduce the incidence of foodborne disease cases.

The bacterial cell envelope provides structural integrity to the cell, but also protects these organisms from unpredictable or sometimes hostile environments (Silhavy et al., 2010). Maintaining the integrity and function of the cell envelope under fluctuating environmental conditions is essential for bacterial survival. Bacteria can sense and respond to cell envelope stressors through alternative sigma factors and/or two-component systems (TCS) (Jordan et al., 2008). *L. monocytogenes* harbors at least 15 TCS, four of which have been reported to play a critical role in regulating the cell envelope stress response (*liaRS*, *lisRK*, *cesRK*, and *virRS*) (Nielsen et al., 2012; Kang et al., 2015). A number of genes associated with resistance to nisin and other CAMPs are a part of TCS regulons, including *dltABCD* (Kramer et al., 2008; McBride and Sonenshein, 2011a) and *mprF* (Staubitz and Peschel, 2002; Thedieck et al., 2006; Samant et al., 2009). The gene products of the *dltABCD* operon modify the cell wall by the addition of D-alanine to lipoteichoic acids (LTAs), while the MprF protein modifies the cell membrane by the addition of lysine to phospholipids, resulting in a net positive charge of the cell envelope (Fischer, 1988; Peschel et al., 2001; Thedieck et al., 2006). Mutants lacking these modifications allow for increased binding of CAMPs. For example, in some Gram-positive bacteria, mutations in the *dlt* operon cause the strain to be more sensitive to nisin and other CAMPs such as polymyxin B and gallidermin, presumably due to the lack of D-alanine on the LTAs and the creation of an overall negatively charged cell wall (Kramer et al., 2008; McBride and Sonenshein, 2011a, b).

The environment in which bacteria are grown can affect the efficacy of CAMPs (Jordan et al., 2008; Bergholz et al., 2012, 2013; Nielsen et al., 2012; Kang et al., 2015). For example, exposure of *L. monocytogenes* to salt (NaCl) (Bergholz et al., 2013) and pre-exposure of *L. monocytogenes* to the organic acid potassium lactate (Kang et al., 2015) increased subsequent resistance to nisin. Furthermore, the antimicrobial effect of nisin varies with the food matrix and under different environmental conditions (Jung et al., 1992; Davies et al., 1997; Chen and Hoover, 2006; Khan and Oh, 2016).

Given that the characteristics of Hispanic fresh-style cheese allow for *L. monocytogenes* growth, the objective of this study was to investigate the effects of temperature and pH on nisin treatment efficacy against *L. monocytogenes* in a lab-scale cheese model. Understanding the effects of the environment on antimicrobial treatments will allow for development and application of new strategies or optimization of current control strategies to prevent *Listeria*-related foodborne outbreaks and infections.

## Materials and Methods

### Bacterial Strains and Growth Conditions

Four recent outbreak strains, encompassing the *L. monocytogenes* serotypes most commonly associated with human clinical cases (4b, 1/2a and 1/2b), and the reference strain 10403S were used in this study (Table 1). To assess the role of cell wall modification genes on the effect of environmental conditions over nisin efficacy, a non-polar, in-frame deletion mutation of *mprF* was constructed from the *L. monocytogenes* 10403S parent strain using the splicing by overlap extension (SOE) method as previously described (Horton et al., 1990; Wiedmann et al., 1998). Additionally, a Δ*dltAmprF* mutant was constructed by deleting the *mprF* open reading frame (ORF) from a Δ*dltA* mutant strain (Tokman et al., 2016) using SOE. All mutations were confirmed by PCR and sequencing of the chromosomal copy of the deletion allele. Strains were streaked from frozen brain heart infusion (BHI; Difco, Becton Dickinson and Co., Sparks, MD, United States) stocks, stored at −80°C in 15% glycerol, onto a BHI agar plate, followed by incubation at 37°C for 24 h. A single colony was subsequently inoculated into 5 mL of BHI broth in 16 mm tubes, followed by incubation at 37°C with shaking (230 rpm) for 16 h (Series 25 Incubator, New Brunswick Scientific, Edison, NJ, United States). After 16 h, 50 μL BHI culture was inoculated into fresh 5 mL BHI broth and grown at 30°C until it reached an OD_600_ = 1.0.

**TABLE 1:** Strains and plasmids used in this study.

**Strains and plasmids**	**Previous ID/description**	**Serotype**	**References**	**Nisin MIC (mg/mL)^1^**
**Strains *L. monocytogenes***				
FSL X1-0001	10403S	1/2a	Bishop and Hinrichs, 1987	0.016
FSL R9-5621	SAMN02566964	1/2a	CDC, 2012	0.016
FSL R9-5623	SAMN02265450	4b	CDC, 2013	0.016
FSL R9-5624	SAMN02689015	1/2b	CDC, 2014	0.031
FSL R9-5625	SAMN02950474	4b	CDC, 2015	0.031
FSL D4-0041	10403S Δ*dltA*	1/2a	Tokman et al., 2016	0.008
FSL B2-0451	10403S Δ*mprF*	1/2a	This study	0.004
FSL B2-0445	10403S Δ*dltAmprF*	1/2a	This study	0.004
***P. cerevisiae***				
FSL C8-0053	*P. cerevisiae* E66	N/A	Gift from Randy Worobo (Cornell University)	0.004
**Plasmids**				
pBMB100	Δ*mprF* on pKSV7	N/A	This study	
pKSV7	Integrative shuttle vector	N/A	(Smith and Youngman, 1992)	

*^1^Minimal inhibitory concentration (MIC) from triplicate plates. MIC reported as the lowest concentration of nisin that inhibited visible growth (Wiegand et al., 2008).*

### Minimal Inhibitory Concentration Assay

The broth microdilution method described by Van Tassell et al., 2015 was used to measure the minimal inhibitory concentration (MIC) of nisin for all strains of *L. monocytogenes* (wildtype, cheese outbreak isolates, and cell wall mutants), as well as the *Pediococcus cerevisiae* E66 (nisin sensitive strain) as a reference. Overnight cultures were inoculated at approximately 10^5^ cfu/mL into 96-well microtiter plates containing serial 2-fold dilutions of nisin in BHI broth starting at 5 mg/mL. Plates were prepared in triplicate and OD_600_ measurements were made in a Synergy H1 Hybrid plate reader (BioTek, Winooski, VT, United States). The MIC was recorded as the lowest concentration of nisin that inhibited visible growth (Wiegand et al., 2008) after a 24 h incubation period at 37°C, and it is reported in Table 1.

### Growth of *L. monocytogenes* in a Lab-Scale Cheese Model Containing Nisin

To test the effect of dairy-relevant conditions on the efficacy of nisin treatment against *L. monocytogenes*, different pH and temperatures were selected to represent the range of pH and temperature that can be found in cheese environments (e.g., pH of different types of cheese as well as temperatures used during cheese make, aging, and storage). We used a method previously described (Henderson et al., 2019) to make approximately 10 g miniature cheese at different pH (6.5, 6.0, or 5.5) and stored at different temperatures (6, 14, or 22°C). Bacteria, including *L. monocytogenes*, present in the milk before cheese was made, were quantified by plating milk samples on plate count agar (PCA; Difco, Becton Dickson and Co.) and on *L. monocytogenes* plating medium (LMPM; Difco, Becton Dickson and Co.). After 48 h of incubation at 32°C for PCA plates and 24 h of incubation at 30°C for LMPM plates, colonies (if present) were counted using a Q Count Colony Counter (Advanced Instruments, Norwood, MA, United States). No *L. monocytogenes* colonies were found (data not shown).

For treated cheese, 50 mg Nisaplin^TM^ (Danisco) was added to 600 mL of pasteurized, whole milk (equivalent to 2 μg/mL or 2 ppm nisin, since Nisaplin contains 2.5% nisin), prior to acidification, to ensure even distribution throughout the final product (Fowler and McCann, 1971). Immediately after cheese was made, it was surface inoculated with 100 μL of a stationary phase (OD_600_ = 1.0) culture of *L. monocytogenes* for a target level of inoculation of approximately 10^7^ cfu/g. For pH and temperature experiments, four selected *L. monocytogenes* outbreak strains were used (Table 1). For cell wall mutant experiments, three *L. monocytogenes* 10403S isogenic deletion mutants (Δ*dltA*, Δ*mprF*, and Δ*dltAmprF*) were used (Table 1). Plates of six cheese per plate were covered and incubated at 6, 14 or 22°C for 1, 7, or 14 days. For cell wall deletion mutant experiments, cheese was incubated at 6°C for 1 day. All plates included a lab strain (10403S), as well as an un-inoculated cheese control.

On the day of sampling, cheese was diluted 1/10 with PBS and homogenized using a Stomacher (Seward., Worthing, United Kingdom). Homogenates were then serially diluted and plated on modified Oxford agar (MOX; Difco, Becton Dickinson and Co.) using an Autoplate spiral plating system (Advanced Instruments., Norwood, MA, United States). After 48 h of incubation at 30°C (Curtis et al., 1989), *L. monocytogenes* colonies were quantified using a Q Count Colony Counter (Advanced Instruments). Experiments were performed in biological triplicate.

### Nisin Extraction and Activity Assay

To evaluate whether a different amount of nisin was lost during preparation of cheese at different pH, nisin was extracted from cheese using a modified acid extraction method as previously described (Bouksaim et al., 2000). While 2 ppm nisin was enough to inhibit *L. monocytogenes* growth on our cheese experiments, it was an insufficient amount to extract from cheese; therefore, for nisin extraction experiments, cheese was made with 25 ppm nisin at each pH (6.5, 6.0, or 5.5). To extract nisin, each 10 g cheese was added to 40 mL 0.02 N HCl. The pH was then adjusted to 2.0 with 6 N HCl and the samples were heated to 100°C for 5 min. Samples were then cooled to 20°C and the volume was adjusted to 40 mL with 0.02 N HCl. Samples were centrifuged for 20 min at 4000 × *g* at 4°C. The supernatant was held at 4°C for 30 min and then filtered through a 0.22 μm sterile filter. The extracts were adjusted to pH 5.5 using 6 N NaOH to ensure high nisin solubility and stored at 4°C until nisin activity testing.

For nisin activity assessment, a well agar diffusion method (Balouiri et al., 2016) was used in which wells (8.8 mm in diameter) were cut out of De Man, Rogosa and Sharpe (MRS) (Difco, Becton Dickinson and Co.) agar plates, and a total volume of 500 μL of nisin extracted from the cheese was added to each well. A control well was made using 500 μL of a 25 ppm nisin stock preparation. The nisin was allowed to diffuse into the agar, then 6 mL of MRS soft agar (0.75%) seeded with ∼10^5^ cfu/mL of *P. cerevisiae* E66 (nisin sensitive strain) was overlaid onto the plates. After the agar solidified, the plates were incubated at 30°C for 24 h. The diameter of zones of inhibition were measured to determine nisin activity. Experiments were performed as two independent biological replicates.

### Statistical Analysis

All statistical analyses were carried out in R Statistical Programming Environment (R Core Team, 2015). We constructed individual linear mixed effects models for temperature and pH using “lmer” function in the “lme4” package (Bates et al., 2015). For each model, the response was the log cfu/g of the number of *L. monocytogenes*, defined as log count and random effects were (i) replicate and (ii) plate nested within cheese make and milk batch. Fixed effects were (i) temperature or pH, (ii) day, (iii) nisin, (iv) strain, (v) age of the milk (based on a 21-day code date), (vi) log of the aerobic plate counts (bacterial counts in the milk before cheese was made; milk apc), and (vii) inoculum (log cfu/g of *L. monocytogenes* inoculated on each cheese). We also included interactions between (i) nisin and temperature or pH, (ii) nisin and strain, and (iv) nisin and day. To address multicollinearity, variance inflation factors were investigated and brought into an acceptable range, resulting in the exclusion of “age of the milk” from both the temperature model and the pH model (Zuur et al., 2010). To address an issue of singularity for the temperature model, the random effect of “replicate” was excluded. To address a model convergence issue, a single outlier was identified and removed from the pH model. Model assumptions were verified for the final version of both the temperature model and the pH model.

A one-way analysis of variance (ANOVA) was calculated for the effect of pH on nisin extracted from cheese. A two-way ANOVA was calculated for the effect of strain and pH on *L. monocytogenes* log reduction for experiments using the mutant strains; *post hoc* analysis was performed using Tukey’s honestly significant difference. The cut-off for significance for all statistics was set at *P* < 0.05. Raw data and the R code used for all statistical analyses are available on GitHub^1^.

## Results

### Temperature Affects *L. monocytogenes’* Susceptibility to Nisin

**FIGURE 1:**
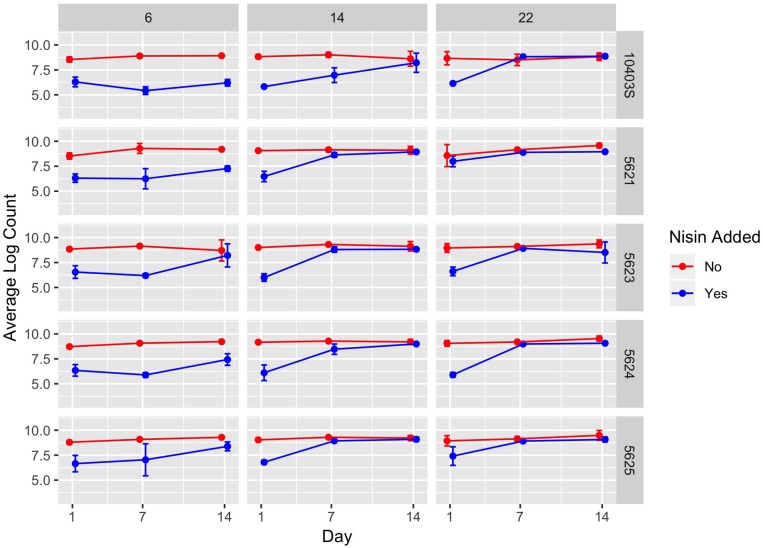
Average counts (log cfu/g) of *L. monocytogenes* in the presence (blue line) and absence (red line) of nisin treatment in a lab-scale cheese model. Each cheese was inoculated with a single strain of *L. monocytogenes* (10403S, FSL R9-5621, FSL R9-5623, FSL R9-5624, or FSL R9-5625) to a level of approximately 7 log cfu/g. These results represent the effect of temperature (6, 14, and 22°C) on *L. monocytogenes*’ sensitivity to nisin. The data points are slightly offset such that the reader can clearly see each point; however, the points still correspond to 1, 7, and 14 days. All values are the arithmetic mean of three independent experiments, and error bars denote standard error. For some data points, error bars are not visible because standard error was too low to yield a visible error bar. All cheese was made at pH 6.5.

**FIGURE 2:**
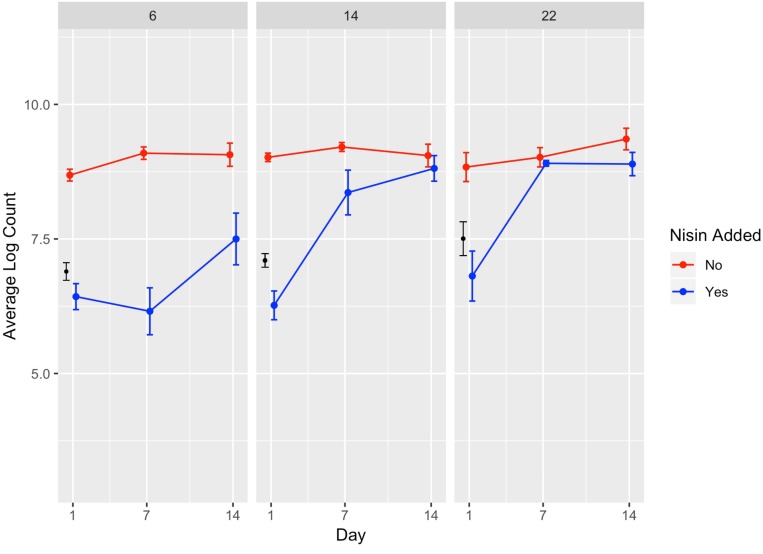
Average counts (log cfu/g) of *L. monocytogenes* in the presence (blue line) and absence (red line) of nisin treatment in a lab-scale cheese model. Calculated initial (day 0) *L. monocytogenes* numbers based on the average inoculum level (approximately 7 log cfu/g) are shown in black. Each cheese was inoculated with a single strain of *L. monocytogenes* (10403S, FSL R9-5621, FSL R9-5623, FSL R9-5624, or FSL R9-5625) to a level of approximately 7 log cfu/g. These results represent the effect of temperature (6, 14, and 22°C) on *L. monocytogenes*’ sensitivity to nisin. The data points are slightly offset such that the reader can clearly see each point; however, the points still correspond to 1, 7, and 14 days. All values are the arithmetic mean of three independent experiments, and error bars denote standard error. All cheese was made at pH 6.5.

Nisin (2 ppm) was added to pasteurized milk prior to production of a lab-scale cheese model (pH 6.5) to assess the effect of different cheese incubation temperatures on the ability of nisin to reduce *L. monocytogenes* numbers on cheese. Cheese was surface inoculated with one of four recent *L. monocytogenes* outbreak strains or reference strain 10403S and then incubated at either 6, 14, or 22°C prior to quantification of *L. monocytogenes* numbers at day 1, 7, and 14. In general, nisin-treated cheese showed lower numbers of *L. monocytogenes* compared to untreated cheese at each temperature tested; however, higher *L. monocytogenes* numbers were observed for nisin-treated cheese stored at 14 and 22°C compared to nisin-treated cheese stored at 6°C. Figure 1 represents the observed data for each strain individually, while Figure 2 shows *L. monocytogenes* counts averaged for all strains. A linear mixed effects model was used to specifically determine whether (i) temperature, (ii) day of incubation, (iii) presence or absence of nisin, and (iv) strain as well as interactions between (v) temperature and nisin, (vi) strain and nisin, and (vii) nisin and day showed significant effects on log transformed bacterial numbers (Table 2).

**TABLE 2:** Model parameters for all fixed effects in the temperature model for *L. monocytogenes* counts.

**Response variable**	**Fixed effects**	**Levels**	**Estimate**	**Standard error**	***P*-value**	**Significance**
*L. monocytogenes* count (log cfu/g)	Temperature (°C)	6	Ref^1^			
		14	0.14	0.22	0.541	
		22	0.10	0.23	0.666	
	Nisin	N^2^	Ref			
		Y^3^	−3.44	0.29	<0.001	***
	Day	1	Ref			
		7	0.26	0.21	0.227	
		14	0.31	0.21	0.151	
	Strain	10403S	Ref			
		5621	0.31	0.12	0.008	**
		5623	0.30	0.12	0.010	*
		5624	0.39	0.12	0.001	**
		5625	0.38	0.12	0.001	**
	Inoculum^4^ (log cfu/g)		0.05	0.11	0.676	
	Milk apc^5^ (log cfu/mL)		0.00	0.09	0.967	
	Temperature:Nisin Y	6:Nisin Y	Ref			
		14:Nisin Y	0.97	0.30	0.002	**
		22:Nisin Y	1.39	0.30	<0.001	***
	Nisin Y:Day	Nisin Y:1	Ref			
		Nisin Y:7	1.05	0.30	0.001	**
		Nisin Y:14	1.59	0.30	<0.001	***
	Nisin Y:Strain	Nisin Y:Strain 10403S	Ref			
		Nisin Y:Strain 5621	0.45	0.16	0.007	**
		Nisin Y:Strain 5623	0.33	0.16	0.043	*
		Nisin Y:Strain 5624	0.08	0.16	0.626	
		Nisin Y:Strain 5625	0.66	0.16	<0.001	***

^1^Ref indicates reference; therefore, estimates, standard error, and *P*-values were not calculated. ^2^N denotes the absence of nisin. ^3^Y denotes the presence of nisin. ^4^Average number (log cfu/g) of *L. monocytogenes* inoculated onto each cheese. ^5^Bacterial aerobic plate counts (apc; log cfu/mL) in milk before cheese was made. *** *P* < 0.001; ** *P* < 0.01; * *P* < 0.05.

A significant effect (*P* < 0.001) on *L. monocytogenes* numbers was found for presence of nisin with a model-estimated effect size of −3.44, indicating 3.44 log cfu/g lower *L. monocytogenes* numbers in the presence of nisin (Table 2). The significance of the factor “nisin” supports that nisin significantly reduces *L. monocytogenes* numbers in the cheese model, as evidenced by the data shown in Figures 1, 2. For cheese incubated at 6°C, average *L. monocytogenes* numbers (across all sampling days and strains) were 6.69 and 8.95 log cfu/g for cheese made with and without nisin, respectively (Supplementary Table 1). Nisin-treated cheese also consistently showed lower bacterial numbers at day 1 as compared to untreated cheese (2.26, 2.78, and 2.03 log cfu/g lower average *L. monocytogenes* numbers [across the 5 strains tested] for 6, 14, and 22°C) (Figure 2, Supplementary Table 1).

The interaction between nisin and day 7 and 14 also had significant effects on *L. monocytogenes* numbers with higher numbers at both days as compared to the reference (i.e., day 1) (Table 2). The significance of this interaction is not surprising considering that *L. monocytogenes* showed growth over time even in the presence of nisin (average log counts (cfu/g) across strains were 6.4 and 7.5 for day 1 and 14, respectively, at 6°C; Figure 2, Supplementary Table 1).

We also found a significant interaction effect between nisin and storage at 14 and 22°C with an effect size of 0.97 and 1.39, respectively, indicating 0.97 and 1.39 log cfu/g higher *L. monocytogenes* numbers relative to nisin treatment at 6°C (Table 2). While on average, nisin-treated cheese showed lower *L. monocytogenes* numbers across all temperatures, the difference between *L. monocytogenes* numbers on treated and untreated cheese varied considerably by temperature (Figure 2). For example, the lowest log difference between nisin-treated and untreated cheese for day 1 (0.58 log) was found for strain 5621 grown on cheese incubated at 22°C, with higher corresponding log differences of 2.22 and 2.59 for 6 and 14°C, respectively (Figure 1 and Supplementary Table 1). Importantly, however, growth of *L. monocytogenes* was still observed in nisin-treated cheese, particularly those stored at 14 and 22°C (Figures 1, 2) where, for a number of strains, by day 7 and 14, *L. monocytogenes* numbers were similar for nisin-treated and untreated cheese and differed by <0.5 log. For example, at 6°C, only strains 10403S, 5621, and 5624 (both day 7 and 14) showed >0.5 log difference between nisin-treated and untreated cheese.

Lastly, we found a significant interaction effect between presence of nisin and strains 5621 (serotype 1/2a), 5623 (serotype 4b), and 5625 (serotype 4b) (*P* = 0.007; effect size of 0.45, *P* = 0.043; effect size of 0.33 and *P* < 0.001; effect size of 0.66, respectively) (Table 2). This indicates that these strains have reduced sensitivity to nisin with 0.45, 0.33, and 0.66 log cfu/g higher *L. monocytogenes* numbers in the presence of nisin as compared to the reference strain 10403S. While strain 5625 showed a higher broth MIC compared to 10403S (Table 1), strains 5621 and 5623 did not.

### pH Affects *L. monocytogenes’* Susceptibility to Nisin Treatment

**FIGURE 3:**
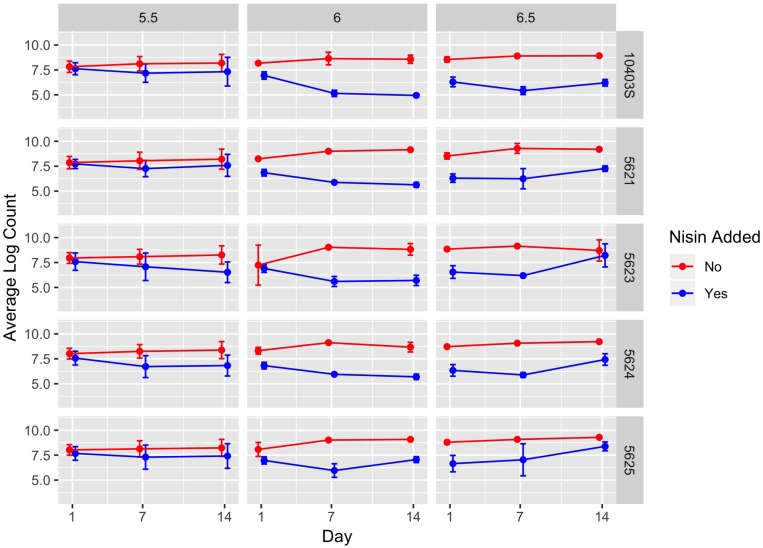
Average counts (log cfu/g) of *L. monocytogenes* in the presence (blue line) and absence (red line) of nisin treatment in a lab-scale cheese model. Each cheese was inoculated with a single strain of *L. monocytogenes* (10403S, FSL R9-5621, FSL R9-5623, FSL R9-5624, or FSL R9-5625) to a level of approximately 7 log cfu/g. These results represent the effect of pH (5.5, 6.0, and 6.5) on *L. monocytogenes*’ sensitivity to nisin. The data points are slightly offset such that the reader can clearly see each point; however, the points still correspond to 1, 7, and 14 days. All values are the arithmetic mean of three independent experiments, and error bars denote standard error. For some data points, error bars are not visible because standard error was too low to yield a visible error bar. All cheese was stored at 6°C.

**FIGURE 4:**
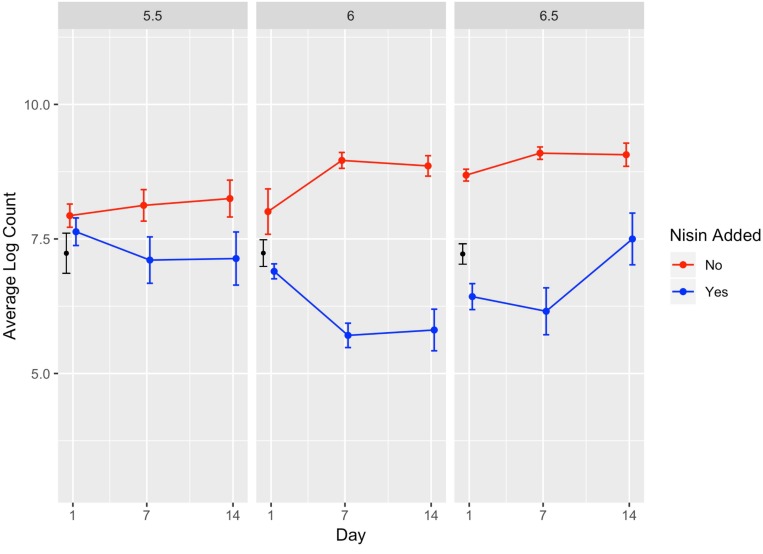
Average counts (log cfu/g) of *L. monocytogenes* in the presence (blue line) and absence (red line) of nisin treatment in a lab-scale cheese model. Calculated initial (day 0) *L. monocytogenes* numbers based on the average inoculum level (approximately 7 log cfu/g) are shown in black. Each cheese was inoculated with a single strain of *L. monocytogenes* (10403S, FSL R9-5621, FSL R9-5623, FSL R9-5624, or FSL R9-5625) to a level of approximately 7 log cfu/g. These results represent the effect of pH (5.5, 6.0, and 6.5) on *L. monocytogenes*’ sensitivity to nisin. The data points are slightly offset such that the reader can clearly see each point; however, the points still correspond to 1, 7, and 14 days. All values are the arithmetic mean of three independent experiments, and error bars denote standard error. All cheese was stored at 6°C.

Nisin (2 ppm) was added to the milk, and cheese was made at pH 5.5, 6.0, and 6.5 to assess the effect of different pH on nisin inhibition of *L. monocytogenes*. Bacterial numbers were quantified after storage at 6°C for 1, 7, and 14 days (Figure 3). In general, nisin-treated cheese showed lower numbers of *L. monocytogenes* compared to untreated cheese at each pH tested; however, higher *L. monocytogenes* numbers were observed for nisin-treated cheese made at pH 5.5 compared to nisin treated cheese made at pH 6.0 and 6.5. Figure 3 represents the observed data for each strain individually, while Figure 4 shows *L. monocytogenes* counts averaged for all strains. A linear mixed effects model was used to determine whether (i) pH, (ii) day of incubation, (iii) presence or absence of nisin, and (iv) strain as well as interactions between (v) pH and nisin, (vi) strain and nisin, and (vii) nisin and day showed significant effects on log transformed bacterial numbers (Table 3).

**TABLE 3:** Model parameters for all fixed effects in the pH model for *L. monocytogenes* counts.

**Response variable**	**Fixed effects**	**Levels**	**Estimate**	**Standard error**	***P*-value**	**Significance**
*L. monocytogenes* count (log cfu/g)	pH	6.5	Ref^1^			
		6.0	−0.28	0.25	0.270	
		5.5	−0.83	0.26	0.003	**
	Nisin	N^2^	Ref			
		Y^3^	−1.89	0.33	<0.001	***
	Day	1	Ref			
		7	0.46	0.25	0.074	
		14	0.45	0.25	0.075	
	Strain	10403S	Ref			
		5621	0.19	0.11	0.081	
		5623	0.13	0.11	0.229	
		5624	0.22	0.11	0.045	*
		5625	0.21	0.11	0.052	
	Inoculum^4^ (log cfu/g)		−0.02	0.08	0.838	
	Milk apc^5^ (log cfu/mL)		−0.02	0.11	0.859	
	pH:Nisin Y	6.5:Nisin Y	Ref			
		6.0:Nisin Y	−0.28	0.35	0.432	
		5.5:Nisin Y	1.44	0.35	< 0.001	***
	Nisin Y:Day	Nisin Y:1	Ref			
		Nisin Y:7	−1.12	0.35	0.003	**
		Nisiin Y:14	−0.63	0.35	0.082	
	Nisin Y:Strain	Nisin Y:10403S	Ref			
		Nisin Y:5621	0.23	0.15	0.128	
		Nisin Y:5623	0.24	0.15	0.111	
		Nisin Y:5624	0.03	0.15	0.834	
		Nisin Y:5625	0.61	0.15	<0.001	***

^1^Ref indicates reference; therefore, estimates, standard error, and *P*-values were not calculated. ^2^N denotes the absence of nisin. ^3^Y denotes the presence of nisin. ^4^Average number (log cfu/g) of *L. monocytogenes* inoculated onto each cheese. ^5^Bacterial aerobic plate counts (apc; log cfu/mL) in milk before cheese was made. *** *P* < 0.001; ** *P* < 0.01; * *P* < 0.05

A significant effect (*P* < 0.001) was found for presence of nisin with an effect size of −1.89, which indicates *L. monocytogenes* numbers are 1.89 log cfu/g lower in the presence of nisin treatment (Table 3). For cheese made at pH 6.0, average bacterial numbers (across all sampling days and strains) were 6.14 and 8.61 log cfu/g for cheese made with and without nisin, respectively (Figure 4 and Supplementary Table 1). Although nisin-treated cheese showed lower *L. monocytogenes* numbers across pH, the difference between *L. monocytogenes* numbers on treated and untreated cheese varied considerably by pH; for example, the lowest log difference between nisin-treated and untreated cheese for day 1 (0.14 log) was found for strain 5621 on cheese made at pH 5.5, with higher corresponding log differences of 1.39 and 2.22 for pH 6.0 and 6.5, respectively (Figure 3 and Supplementary Table 1). Differences between *L. monocytogenes* numbers on treated and untreated cheese made at pH 5.5 were minimal and ranged from 0.14 to 1.73 log cfu/g, based on observed data across strains as compared to 0.33–3.63 log cfu/g and 0.49–3.47 log cfu/g for pH 6.0 and pH 6.5, respectively (Supplementary Table 1 and Figure 4). Similar to results obtained from the temperature model, *L. monocytogenes* still grew in nisin-treated cheese, but only in cheese made at pH 6.5 (Figure 3) where, by day 14, all *L. monocytogenes* strains had higher numbers for nisin-treated cheese compared to day 7.

We found a significant interaction effect between nisin and pH 5.5 with an effect size of 1.44, indicating 1.44 log cfu/g higher *L. monocytogenes* numbers relative to nisin treatment at pH 6.5 (Table 3). The fact that nisin seemed less effective against *L. monocytogenes* in cheese made at pH 5.5 is surprising considering that nisin is more stable at lower pH (Tan et al., 2015); thus, one could have hypothesized that nisin would have effectively killed *L. monocytogenes* at this pH.

We also found significant interaction effects between presence of nisin and strain 5625, one of the two 4b strains used here (*P* < 0.001; effect size 0.61) (Table 3); this indicates that this strain shows 0.61 log cfu/g higher numbers in the presence of nisin as compared to the reference strain 10403S. Strain 5625, as mentioned above, also showed a higher MIC in broth compared to 10403S (Table 1).

### *L. monocytogenes* Reduced Sensitivity to Nisin on Lab-Scale Cheese Made at pH 5.5 Is at Least Partially *dltA*- and *mprF*-Dependent

As mentioned before, the reduced nisin sensitivity effect observed for strains growing in cheese made at pH 5.5 was surprising given the fact that nisin should be stable at this pH (Tan et al., 2015). We hypothesized that this effect could be due to (i) a more pronounced loss of nisin in the whey for cheese made at pH 5.5 compared to pH 6.5 as nisin is more soluble at lower pH (Liu and Hansen, 1990) or (ii) a response in cells grown at pH 5.5 that involves the nisin resistance genes *dltA* and *mprF*.

**FIGURE 5:**
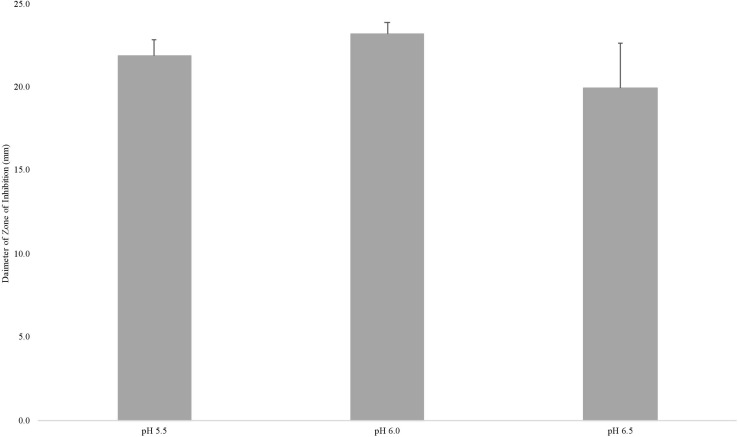
Average size (mm) of zone of inhibitions of nisin extracted from cheese made at pH 5.5, 6.0, and 6.5 against *P. cerevisiae*. Results are of two independent biological replicates. Error bars denote standard error.

To test whether nisin loss was responsible for the reduced sensitivity of *L. monocytogenes* to nisin on cheese made at pH 5.5, 25 ppm nisin was added to the milk, and cheese was made at pH 5.5, 6.0, and 6.5. After extracting nisin from each cheese, soft agar diffusion assays were performed using the nisin sensitive strain *P. cerevisiae* E66 and the diameter of the zones of inhibition caused by the extracted nisin were measured (Figure 5). A one-way ANOVA was used to determine differences in zones of inhibition between the nisin-extracted samples; the results show there was no significant difference among diameters of the zones of inhibition (*P* = 0.31; Supplementary Table 2), indicating that nisin loss in the whey is similar regardless of the pH (5.5, 6.0, or 6.5) at which the cheese was made, therein nisin loss does not explain the decreased ability of nisin to kill *L. monocytogenes* in cheese made at pH 5.5.

**FIGURE 6:**
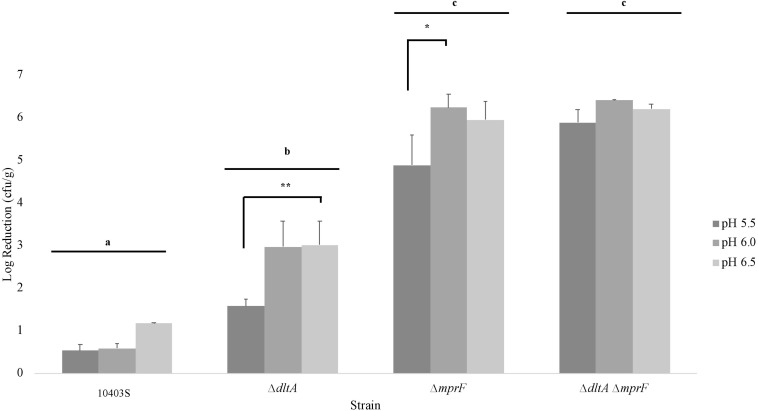
Log reduction of *L. monocytogenes* strains on cheese made at pH 5.5, 6.0, and 6.5 between untreated and treated cheese. These results represent the effect of pH on *L. monocytogenes*’ (10403S, Δ*dltA*, Δ*mprF*, and Δ*dltAmprF*) sensitivity to nisin. Bar groups (i.e., 10403S, Δ*dltA*, Δ*mprF*, and Δ*dltAmprF*) that do not share any letters represent values that are significantly different between strains. Asterisks denote a significant difference between pH 5.5 and pH 6.0 or 6.5 within a strain. Results are an average of three biological replicates. The log reduction was calculated separately for each replicate and then averaged. Error bars denote standard error.

To determine whether the significant interaction effect between nisin and pH 5.5 involves the activity of nisin resistance genes (*dltA* and *mprF*), nisin (2 ppm) was added to the milk, and cheese was made at pH 5.5, 6.0, and 6.5 prior to *L. monocytogenes* (10403S, Δ*dltA*, Δ*mprF*, and Δ*dltAmprF*) surface inoculation. Bacterial numbers were quantified after storage at 6°C for 1 day and the log reduction (between untreated cheese and nisin-treated cheese) was calculated (Figure 6). A two-way ANOVA was calculated for the effect of strain and pH on *L. monocytogenes* log reduction and *post hoc* analysis was performed using Tukey’s HSD (Supplementary Tables 3, 4, respectively).

As expected, the cell wall mutant strains Δ*dltA*, Δ*mprF*, and Δ*dltAmprF* showed an increased sensitivity to nisin, consistent with their lower broth MIC (Table 1), compared to the parental strain 10403S (Figure 6). Interestingly, pH 5.5 still led to significantly reduced nisin sensitivity in the Δ*dltA* and Δ*mprF* single mutants, when compared to pH 6.0, and in the Δ*dltA* when compared to pH 6.5, however, this was not observed in the double mutant Δ*dltAmprF*, suggesting that the reduced nisin susceptibility observed at pH 5.5 involves both of these genes (Figure 6 and Supplementary Table 4).

## Discussion

Overall, our results indicate that nisin can reduce *L. monocytogenes* numbers on a lab-scale cheese model, however, *L. monocytogenes* growth was still observed, even in the presence of nisin. Furthermore, pH, temperature, and strain serotype showed significant effects on the efficacy of nisin treatment. These findings highlight the need to consider environmental conditions, including those specific to the product (e.g., pH), to optimize the effectiveness of nisin treatment on foods. While an initial log reduction, as that we observed with nisin, may reduce the overall risk of human listeriosis cases linked to fresh cheese, a formal risk assessment considering several of these parameters would be needed to more precisely assess the public health impact of nisin application on cheese. Importantly, a previous risk assessment focused on surface ripened soft raw milk cheeses did, however, suggest that even log reductions less than 5 log can have a considerable public health impact with regard to human listeriosis cases in the US (FDA, 2015).

### Nisin Efficacy Is Enhanced at Lower Storage Temperatures

Our data showed that nisin can significantly decrease *L. monocytogenes* numbers in cheese and that its efficacy is enhanced when cheese is stored at lower temperatures. However, *L. monocytogenes* was able to grow on nisin-treated cheese, and more rapid growth is seen when cheese is stored at higher temperatures.

We found that *L. monocytogenes* numbers were approximately 1 log lower at day 14 on nisin-treated cheese stored at 6°C compared with nisin-treated cheese stored at 14 or 22°C (Figure 2). Others have shown that nisin is more effective at killing *L. monocytogenes* at lower temperatures. For example, Li et al. (2002) reported that *L. monocytogenes* grown in BHI broth at 10°C was more sensitive to nisin than cells grown at 30°C, likely due to membrane modifications in the cells grown at lower temperature, which cause an increase in fluidity (Li et al., 2002). It is therefore possible that *L. monocytogenes* cells growing on cheese at 6°C have increased membrane fluidity that in turns increases sensitivity to nisin as observed in our results.

In addition to the temperature effect observed on nisin efficacy, our data also show that *L. monocytogenes* growth occurs, even in the presence of nisin, and more rapid growth is seen when cheese is stored at higher temperatures. This finding is consistent with other studies that have shown that *L. monocytogenes* can grow in the presence of nisin on cheese (Ferreira and Lund, 1996; Martinez and Rodriguez, 2005). Additionally, a transient bactericidal effect against *L. monocytogenes*, followed by regrowth of cells in food matrices and laboratory media supplemented with nisin has also been previously reported (Davies et al., 1996). There are a number of possible reasons for re-growth of *L. monocytogenes* after an initial significant reduction, including, but not limited to (i) conditions intrinsic to the cheese matrix that decrease nisin availability or activity, and (ii) the physiological state of the cells that could increase the survival rate of the culture. For example, binding of nisin to food matrix components, such as fat (Jung et al., 1992), decrease nisin availability and therefore, its ability to bind and kill *L. monocytogenes*. In addition, nisin is known to be less stable at near neutral pH (such as that of queso fresco) (Gharsallaoui et al., 2016), which could possibly affect its activity in this food matrix. Emergence of nisin resistant cells (Gandhi and Chikindas, 2007) and/or cells that have acclimated to the presence of nisin (Chi-Zhang et al., 2004), could also explain a subsequent regrowth after an initial reduction. It would be interesting to test whether cells recovered from our cheese model made with nisin show any evidence of developing a higher resistance to this bacteriocin after 14 days compared to the original culture. Given the potential for regrowth of *L. monocytogenes* during long-term refrigerated storage, even after an initial reduction due to nisin treatment, studies over product shelf-life that consider storage time and temperature are essential to appropriately assess nisin treatment efficacy and evaluate the usefulness of nisin applications and their impact for public health. In addition, alternate strategies could be used to overcome the limitations of nisin when used in fresh cheese (Ibarra-Sánchez et al., 2020) including nisin encapsulation (Feng et al., 2019) as well as combination with other antimicrobials (Ibarra-Sánchez et al., 2018).

### The Effect of pH 5.5 to Decrease Nisin Efficacy Against *L. monocytogenes* Is Partially Due to the Activity of *dltA* and *mprF*

Our data showed that when cheese is formulated at pH 5.5, nisin is less effective at killing *L. monocytogenes* compared to when cheese is made at pH 6.5. *L. monocytogenes* encounters many stresses in a food environment, and tolerance to a stress condition could lead to cross-protection against a subsequent stress (van Schaik et al., 1999; Bergholz et al., 2013; Kang et al., 2015). Furthermore, transcriptome profiling in *L. monocytogenes* under acid-induced conditions shows up-regulation of the VirR regulon (Tessema et al., 2012), which regulates at least 12 genes, including *dltABCD* and *mprF*, both of which confer resistance to a variety of CAMPs, including nisin. Wall teichoic acids (WTAs) are a main component of the Gram-positive bacterial cell wall and are highly negatively charged due to deprotonized phosphate groups. It has been shown experimentally, that Gram-positive bacteria, such as *Staphylococcus aureus* (Peschel et al., 1999), *Clostridium difficile* (McBride and Sonenshein, 2011b), *Bacillus cereus* (Abi Khattar et al., 2009), and *L. monocytogenes* (Reichmann et al., 2013) can resist interactions with bacteriocins by upregulating the *dlt* operon, which encodes proteins that incorporate D-alanine residues onto teichoic acids, reducing the net negative charge of the cell wall (Fischer, 1988). Additionally, the product of the *mprF* gene is required for the synthesis of lysylphosphatidylglycerol and the addition of L-lysine to phosphatidylglycerol, which also reduce the net negative charge of the cell membrane (Peschel et al., 2001; Thedieck et al., 2006). As bacteriocins are positively charged, the activity of *dltA* and *mprF*, will result in inhibition of nisin action on the cell wall. We hypothesized that the decreased effect of nisin against *L. monocytogenes* in cheese formulated at pH 5.5 is at least partially dependent on the presence of nisin-resistance genes *dltA* and *mprF*. While our results show a greater sensitivity to nisin in Δ*dltA* and Δ*mprF* mutant strains compared to 10403S, pH 5.5 still showed a protective effect in the single mutants but not in the double mutant (Δ*dltAmprF*). Therefore, the reduced sensitivity to nisin at pH 5.5 could be partially due to the activity of *dltA* and *mprF* from upregulation of the VirR regulon at pH 5.5. It would be interesting to test if the VirR regulon, along with *dltA* and *mprF*, is induced in *L. monocytogenes* growing on our cheese model made at pH 5.5.

Reduced sensitivity to nisin, independent of *dltA* and *mprF*, has also been associated with changes in cell membrane fatty acid composition, resulting in more rigid membrane fluidity (Ming and Daeschel, 1993; van Schaik et al., 1999). Changes in cell membrane composition can be attributed to acid tolerance in *L. monocytogenes*, in which diffusion of fatty acids across the membrane is inhibited, resulting in more rigid cell membranes (Beales, 2004), and subsequently, partially decreasing sensitivity to nisin. Furthermore, it has been shown that *L. monocytogenes* uses a glutamate decarboxylase system to survive acid stress (Cotter et al., 2001; Begley et al., 2010; Lourenco et al., 2017). GadD1 catalyzes the breakdown of glutamate into -aminobutyrate and carbon dioxide, forming ATP, which could restore intracellular levels of ATP that are depleted by nisin activity on the cell (van Schaik et al., 1999), and thus lead to reduced sensitivity to nisin. It is likely that the increased resistance to nisin observed in *L. monocytogenes* growing on cheese made at pH 5.5 is a result of a combination of processes including *dltA*- and *mprF*- mediated modifications of the cell membrane, changes in membrane fluidity, and acid tolerance. Overall, our data suggest that pH can significantly affect the efficacy of nisin against *L. monocytogenes* on cheese, therefore, the pH of cheese should be considered for *L. monocytogenes* control strategies, especially given the range in pH of different cheese types. Our results also highlight the importance of considering the intrinsic characteristics of a product when assessing the efficacy of *L. monocytogenes* control strategies. Follow up studies would be needed to explore the role of other intrinsic cheese characteristics, such as salt concentration, on nisin treatment on cheese.

### *L. monocytogenes* Serotype Affects Nisin Efficacy

In addition to the effects of environmental conditions, our data also indicate that both serotype 4b strains showed reduced sensitivity to nisin across temperature and that serotype 4b strain 5625 also showed reduced sensitivity to nisin across pH. Interestingly, while strain 5625 (serotype 4b) showed a higher MIC in broth compared to the reference strain 10403S, strain 5623 (serotype 4b) did not (Table 1), which again highlights the importance of considering the environmental conditions specific to the food matrix when studying the efficacy of an antimicrobial strategy. Our findings are consistent with previous work that shows that the *L. monocytogenes* Scott A (4b) strain appeared to be more resistant to nisin compared to strains other serotypes (Ukuku and Shelef, 1997). However, others have shown no correlation between the differences in nisin sensitivity and serotype (Ferreira and Lund, 1996; Martinez and Rodriguez, 2005). In contrast, Buncic et al., 2001, showed that serotype 1/2a isolates were more resistant to two antilisterial bacteriocins (Lb 265 and Lb706) than serotype 4b isolates (Buncic et al., 2001). More recently, a screen of 282 *L. monocytogenes* isolates from German RTE food products and food-processing environments revealed various degrees of nisin sensitivity between strains, however, isolates derived from milk/cheese and other dairy products (which were predominantly serotype 1/2a) showed significantly higher resistance to nisin concentrations than isolates from other sources (*p* < 0.002) (Szendy et al., 2019). It seems plausible that the overrepresentation of isolates from dairy products with higher resistance to bacteriocins could be explained by the intrinsic advantage these isolates would have in environments where bacteriocin-producing organisms are found.

Our data are also consistent with a previous study that found that serotype also affects the effectiveness of phage treatment against *L. monocytogenes* in a lab-scale cheese model (Henderson et al., 2019) where the same 4b strains used in this study were also more resistant to phage treatment. Although, different serotypes (1/2a, 1/2b, and 4b) have been linked to a number of Hispanic-style cheese outbreaks (CDC, 2012, 2013, 2014, 2015, 2017), historically, isolates of serotype 4b strains have caused the greatest proportion of listeriosis outbreaks and the largest number of outbreak-associated cases compared to serotype 1/2a and 1/2b strains (Cartwright et al., 2013). Differences in nisin sensitivity among *L. monocytogenes* serotypes highlight the importance of validating nisin-based treatment strategies using different *L. monocytogenes* serotypes and environmental conditions relevant to a given application.

## Conclusion

This study shows the critical role of temperature, pH, and *L. monocytogenes* serotype can have over the efficacy of antimicrobials intended for food preservation. Improved understanding of how environmental conditions affect antimicrobial efficacy could facilitate the development and/or effective implementation of control strategies. Additionally, the potential for cross-protection induced by food-relevant stress (e.g., pH) should be considered to avoid overestimation of antimicrobial strength in a food product. Our data suggest that nisin-based control strategies could be successful when cheese is formulated at near neutral pH and stored at low temperature (6°C). However, it is likely that other intrinsic characteristics of cheese (e.g., salt concentration) may also affect nisin efficacy against *L. monocytogenes* on this food matrix.

## Data Availability Statement

All datasets generated for this study are included in the article/Supplementary Material.

## Author Contributions

LH, BE, and JS performed experiments. LH, DK, and SM performed statistical analysis. MW and VG-O conceived the study. LH, MW, and VG-O wrote the manuscript. All authors read and approved the final manuscript.

## Conflict of Interest

The authors declare that the research was conducted in the absence of any commercial or financial relationships that could be construed as a potential conflict of interest.

## Footnotes

1 https://github.com/lohenderson/Nisin_Listeria_Analysis_Model/
