# Supplementary material for: Nevertheless, She Resisted – Role of the Environment on Listeria monocytogenes Sensitivity to Nisin Treatment in a Laboratory Cheese Model
Source: Front Microbiol. 2020 Apr 9;11:635. doi: 10.3389/fmicb.2020.00635 (PMC7160321; doi:10.3389/fmicb.2020.00635)
Supplement: Supplementary file 1 [file Data_Sheet_1.docx]

**Supplemental Table 1**. Observed *L. monocytogenes* counts on cheese in the presence and absence of nisin.

| Temperature (°C) | pH | Strain | Nisin | Day | Rep^1^ 1 | Rep 2 | Rep 3 | Average^2^ | Std Dev^3^ | Log Reduction (N-Y) |
| --- | --- | --- | --- | --- | --- | --- | --- | --- | --- | --- |
| 6 | 6.5 | 10403S | N | 1 | 8.50 | 8.79 | 8.35 | 8.55 | 0.22 | 2.25 |
| 6 | 6.5 | 5621 | N | 1 | 8.38 | 8.83 | 8.36 | 8.52 | 0.27 | 2.22 |
| 6 | 6.5 | 5623 | N | 1 | 8.92 | 8.95 | 8.66 | 8.85 | 0.16 | 2.30 |
| 6 | 6.5 | 5624 | N | 1 | 8.79 | 8.81 | 8.57 | 8.72 | 0.13 | 2.38 |
| 6 | 6.5 | 5625 | N | 1 | 8.89 | 8.89 | 8.59 | 8.79 | 0.17 | 2.14 |
| 6 | 6.5 | 10403S | Y | 1 | 6.24 | 5.91 | 6.74 | 6.30 | 0.42 |  |
| 6 | 6.5 | 5621 | Y | 1 | 6.02 | 6.17 | 6.72 | 6.31 | 0.37 |  |
| 6 | 6.5 | 5623 | Y | 1 | 6.02 | 6.49 | 7.12 | 6.55 | 0.55 |  |
| 6 | 6.5 | 5624 | Y | 1 | 5.79 | 6.77 | 6.47 | 6.34 | 0.50 |  |
| 6 | 6.5 | 5625 | Y | 1 | 5.84 | 6.96 | 7.16 | 6.65 | 0.71 |  |
| 6 | 6.5 | 10403S | N | 7 | 8.77 | 8.85 | 9.06 | 8.89 | 0.15 | 3.47 |
| 6 | 6.5 | 5621 | N | 7 | 9.78 | 9.02 | 9.05 | 9.28 | 0.43 | 3.04 |
| 6 | 6.5 | 5623 | N | 7 | 9.08 | 9.12 | 9.24 | 9.15 | 0.08 | 2.96 |
| 6 | 6.5 | 5624 | N | 7 | 9.06 | 9.10 | 9.04 | 9.07 | 0.03 | 3.18 |
| 6 | 6.5 | 5625 | N | 7 | 8.99 | 9.21 | 9.03 | 9.08 | 0.12 | 2.04 |
| 6 | 6.5 | 10403S | Y | 7 | 5.12 | 5.78 | 5.36 | 5.42 | 0.33 |  |
| 6 | 6.5 | 5621 | Y | 7 | 5.57 | 5.92 | 7.24 | 6.25 | 0.88 |  |
| 6 | 6.5 | 5623 | Y | 7 | 6.00 | 6.23 | 6.34 | 6.19 | 0.17 |  |
| 6 | 6.5 | 5624 | Y | 7 | 5.69 | 6.11 | 5.86 | 5.89 | 0.21 |  |
| 6 | 6.5 | 5625 | Y | 7 | 6.38 | 6.10 | 8.63 | 7.04 | 1.39 |  |
| 6 | 6.5 | 10403S | N | 14 | 8.95 | 8.80 | 9.00 | 8.92 | 0.10 | 2.71 |
| 6 | 6.5 | 5621 | N | 14 | 9.12 | 9.26 | 9.19 | 9.19 | 0.07 | 1.93 |
| 6 | 6.5 | 5623 | N | 14 | 9.22 | 7.65 | 9.26 | 8.71 | 0.92 | 0.49 |
| 6 | 6.5 | 5624 | N | 14 | 9.22 | 9.19 | 9.25 | 9.22 | 0.03 | 1.79 |
| 6 | 6.5 | 5625 | N | 14 | 9.20 | 9.36 | 9.31 | 9.29 | 0.08 | 0.91 |
| 6 | 6.5 | 10403S | Y | 14 | 6.03 | 6.54 | 6.05 | 6.21 | 0.29 |  |
| 6 | 6.5 | 5621 | Y | 14 | 7.54 | 7.13 | 7.12 | 7.26 | 0.24 |  |
| 6 | 6.5 | 5623 | Y | 14 | 7.92 | 9.33 | 7.40 | 8.21 | 1.00 |  |
| 6 | 6.5 | 5624 | Y | 14 | 7.21 | 8.01 | 7.08 | 7.43 | 0.50 |  |
| 6 | 6.5 | 5625 | Y | 14 | 7.95 | 8.51 | 8.69 | 8.38 | 0.38 |  |
| 6 | 6 | 10403S | N | 1 | 8.14 | 8.10 | 8.28 | 8.83 | 0.17 | 3.00 |
| 6 | 6 | 5621 | N | 1 | 8.11 | 8.25 | 8.36 | 9.06 | 0.08 | 2.59 |
| 6 | 6 | 5623 | N | 1 | 8.15 | 8.34 | 5.23 | 9.00 | 0.03 | 3.01 |
| 6 | 6 | 5624 | N | 1 | 8.61 | 8.32 | 8.02 | 9.16 | 0.11 | 3.06 |
| 6 | 6 | 5625 | N | 1 | 8.58 | 8.24 | 7.40 | 9.03 | 0.16 | 2.24 |
| 6 | 6 | 10403S | Y | 1 | 6.68 | 6.84 | 7.30 | 5.82 | 0.08 |  |
| 6 | 6 | 5621 | Y | 1 | 6.55 | 7.12 | 6.87 | 6.47 | 0.45 |  |
| 6 | 6 | 5623 | Y | 1 | 6.51 | 7.14 | 7.08 | 6.00 | 0.33 |  |
| 6 | 6 | 5624 | Y | 1 | 6.49 | 7.01 | 6.97 | 6.10 | 0.68 |  |
| 6 | 6 | 5625 | Y | 1 | 6.61 | 7.11 | 7.19 | 6.79 | 0.18 |  |
| 6 | 6 | 10403S | N | 7 | 8.85 | 8.02 | 9.04 | 9.01 | 0.22 | 2.04 |
| 6 | 6 | 5621 | N | 7 | 8.90 | 8.92 | 9.17 | 9.15 | 0.17 | 0.51 |
| 6 | 6 | 5623 | N | 7 | 9.01 | 9.00 | 9.07 | 9.31 | 0.04 | 0.51 |
| 6 | 6 | 5624 | N | 7 | 8.94 | 9.22 | 9.20 | 9.28 | 0.08 | 0.81 |
| 6 | 6 | 5625 | N | 7 | 8.94 | 8.93 | 9.16 | 9.29 | 0.03 | 0.36 |
| 6 | 6 | 10403S | Y | 7 | 4.92 | 5.45 | 5.07 | 6.97 | 0.64 |  |
| 6 | 6 | 5621 | Y | 7 | 5.95 | 5.88 | 5.82 | 8.64 | 0.20 |  |
| 6 | 6 | 5623 | Y | 7 | 5.60 | 6.04 | 5.16 | 8.80 | 0.24 |  |
| 6 | 6 | 5624 | Y | 7 | 5.96 | 6.05 | 5.83 | 8.47 | 0.45 |  |
| 6 | 6 | 5625 | Y | 7 | 5.55 | 5.70 | 6.63 | 8.93 | 0.11 |  |
| 6 | 6 | 10403S | N | 14 | 8.39 | 8.34 | 8.98 | 8.62 | 0.64 | 0.41 |
| 6 | 6 | 5621 | N | 14 | 9.07 | 9.26 | 9.12 | 9.10 | 0.34 | 0.16 |
| 6 | 6 | 5623 | N | 14 | 9.13 | 8.23 | 9.06 | 9.13 | 0.41 | 0.30 |
| 6 | 6 | 5624 | N | 14 | 8.72 | 8.23 | 9.10 | 9.19 | 0.22 | 0.20 |
| 6 | 6 | 5625 | N | 14 | 9.09 | 9.04 | 9.10 | 9.22 | 0.22 | 0.13 |
| 6 | 6 | 10403S | Y | 14 | 4.79 | 5.09 | 4.95 | 8.21 | 0.82 |  |
| 6 | 6 | 5621 | Y | 14 | 5.46 | 5.87 | 5.57 | 8.93 | 0.06 |  |
| 6 | 6 | 5623 | Y | 14 | 6.21 | 5.53 | 5.38 | 8.82 | 0.13 |  |
| 6 | 6 | 5624 | Y | 14 | 5.96 | 5.50 | 5.63 | 8.99 | 0.11 |  |
| 6 | 6 | 5625 | Y | 14 | 7.14 | 7.26 | 6.77 | 9.09 | 0.20 |  |
| 6 | 5.5 | 10403S | N | 1 | 8.27 | 7.88 | 7.29 | 7.82 | 0.49 | 0.20 |
| 6 | 5.5 | 5621 | N | 1 | 8.20 | 8.13 | 7.25 | 7.86 | 0.53 | 0.14 |
| 6 | 5.5 | 5623 | N | 1 | 8.38 | 8.01 | 7.46 | 7.95 | 0.47 | 0.36 |
| 6 | 5.5 | 5624 | N | 1 | 8.31 | 8.26 | 7.48 | 8.02 | 0.47 | 0.44 |
| 6 | 5.5 | 5625 | N | 1 | 8.38 | 8.19 | 7.50 | 8.02 | 0.46 | 0.35 |
| 6 | 5.5 | 10403S | Y | 1 | 7.86 | 7.97 | 7.02 | 7.62 | 0.52 |  |
| 6 | 5.5 | 5621 | Y | 1 | 7.89 | 8.01 | 7.26 | 7.72 | 0.40 |  |
| 6 | 5.5 | 5623 | Y | 1 | 7.87 | 8.16 | 6.74 | 7.59 | 0.75 |  |
| 6 | 5.5 | 5624 | Y | 1 | 7.73 | 8.08 | 6.92 | 7.57 | 0.59 |  |
| 6 | 5.5 | 5625 | Y | 1 | 7.83 | 8.16 | 7.02 | 7.67 | 0.59 |  |
| 6 | 5.5 | 10403S | N | 7 | 8.53 | 8.42 | 7.39 | 8.11 | 0.63 | 0.94 |
| 6 | 5.5 | 5621 | N | 7 | 8.63 | 8.30 | 7.20 | 8.04 | 0.75 | 0.78 |
| 6 | 5.5 | 5623 | N | 7 | 8.59 | 8.31 | 7.36 | 8.08 | 0.64 | 1.01 |
| 6 | 5.5 | 5624 | N | 7 | 8.57 | 8.61 | 7.57 | 8.25 | 0.59 | 1.53 |
| 6 | 5.5 | 5625 | N | 7 | 8.56 | 8.51 | 7.32 | 8.13 | 0.70 | 0.83 |
| 6 | 5.5 | 10403S | Y | 7 | 7.37 | 7.85 | 6.30 | 7.17 | 0.80 |  |
| 6 | 5.5 | 5621 | Y | 7 | 7.80 | 7.52 | 6.47 | 7.26 | 0.70 |  |
| 6 | 5.5 | 5623 | Y | 7 | 7.58 | 7.94 | 5.71 | 7.08 | 1.19 |  |
| 6 | 5.5 | 5624 | Y | 7 | 7.01 | 7.50 | 5.66 | 6.72 | 0.95 |  |
| 6 | 5.5 | 5625 | Y | 7 | 7.82 | 7.99 | 6.10 | 7.30 | 1.05 |  |
| 6 | 5.5 | 10403S | N | 14 | 8.71 | 8.52 | 7.33 | 8.19 | 0.75 | 0.86 |
| 6 | 5.5 | 5621 | N | 14 | 8.90 | 8.49 | 7.23 | 8.21 | 0.87 | 0.62 |
| 6 | 5.5 | 5623 | N | 14 | 8.80 | 8.63 | 7.35 | 8.26 | 0.79 | 1.73 |
| 6 | 5.5 | 5624 | N | 14 | 8.87 | 8.74 | 7.52 | 8.38 | 0.74 | 1.55 |
| 6 | 5.5 | 5625 | N | 14 | 8.77 | 8.52 | 7.38 | 8.23 | 0.74 | 0.81 |
| 6 | 5.5 | 10403S | Y | 14 | 8.31 | 7.74 | 5.92 | 7.32 | 1.25 |  |
| 6 | 5.5 | 5621 | Y | 14 | 8.31 | 7.95 | 6.50 | 7.59 | 0.95 |  |
| 6 | 5.5 | 5623 | Y | 14 | 7.07 | 7.01 | 5.49 | 6.53 | 0.90 |  |
| 6 | 5.5 | 5624 | Y | 14 | 7.39 | 7.31 | 5.78 | 6.83 | 0.90 |  |
| 6 | 5.5 | 5625 | Y | 14 | 7.98 | 8.09 | 6.18 | 7.42 | 1.07 |  |
| 14 | 6.5 | 10403S | N | 1 | 9.01 | 8.67 | 8.80 | 8.83 | 0.17 | 3.00 |
| 14 | 6.5 | 5621 | N | 1 | 8.99 | 9.14 | 9.04 | 9.06 | 0.08 | 2.59 |
| 14 | 6.5 | 5623 | N | 1 | 8.98 | 9.04 | 9.00 | 9.00 | 0.03 | 3.01 |
| 14 | 6.5 | 5624 | N | 1 | 9.03 | 9.23 | 9.22 | 9.16 | 0.11 | 3.06 |
| 14 | 6.5 | 5625 | N | 1 | 9.21 | 9.00 | 8.89 | 9.03 | 0.16 | 2.24 |
| 14 | 6.5 | 10403S | Y | 1 |  | 5.77 | 5.88 | 5.82 | 0.08 |  |
| 14 | 6.5 | 5621 | Y | 1 | 6.97 | 6.11 | 6.33 | 6.47 | 0.45 |  |
| 14 | 6.5 | 5623 | Y | 1 | 6.36 | 5.73 | 5.90 | 6.00 | 0.33 |  |
| 14 | 6.5 | 5624 | Y | 1 | 6.88 | 5.72 | 5.71 | 6.10 | 0.68 |  |
| 14 | 6.5 | 5625 | Y | 1 | 6.89 | 6.90 | 6.58 | 6.79 | 0.18 |  |
| 14 | 6.5 | 10403S | N | 7 | 9.25 | 8.96 | 8.83 | 9.01 | 0.22 | 2.04 |
| 14 | 6.5 | 5621 | N | 7 | 9.32 | 8.98 | 9.14 | 9.15 | 0.17 | 0.51 |
| 14 | 6.5 | 5623 | N | 7 | 9.34 | 9.34 | 9.26 | 9.31 | 0.04 | 0.51 |
| 14 | 6.5 | 5624 | N | 7 | 9.36 | 9.28 | 9.20 | 9.28 | 0.08 | 0.81 |
| 14 | 6.5 | 5625 | N | 7 | 9.33 | 9.27 | 9.27 | 9.29 | 0.03 | 0.36 |
| 14 | 6.5 | 10403S | Y | 7 | 7.66 | 6.86 | 6.39 | 6.97 | 0.64 | -1.65 |
| 14 | 6.5 | 5621 | Y | 7 | 8.86 | 8.56 | 8.49 | 8.64 | 0.20 |  |
| 14 | 6.5 | 5623 | Y | 7 | 9.08 | 8.72 | 8.61 | 8.80 | 0.24 |  |
| 14 | 6.5 | 5624 | Y | 7 | 8.92 | 8.46 | 8.03 | 8.47 | 0.45 |  |
| 14 | 6.5 | 5625 | Y | 7 | 8.97 | 8.81 | 9.01 | 8.93 | 0.11 |  |
| 14 | 6.5 | 10403S | N | 14 | 7.90 | 8.82 | 9.14 | 8.62 | 0.64 | 0.41 |
| 14 | 6.5 | 5621 | N | 14 | 9.24 | 8.71 | 9.34 | 9.10 | 0.34 | 0.16 |
| 14 | 6.5 | 5623 | N | 14 | 8.70 | 9.16 | 9.52 | 9.13 | 0.41 | 0.30 |
| 14 | 6.5 | 5624 | N | 14 | 9.12 | 9.02 | 9.44 | 9.19 | 0.22 | 0.20 |
| 14 | 6.5 | 5625 | N | 14 | 8.98 | 9.25 | 9.42 | 9.22 | 0.22 | 0.13 |
| 14 | 6.5 | 10403S | Y | 14 | 8.98 | 8.31 | 7.34 | 8.21 | 0.82 |  |
| 14 | 6.5 | 5621 | Y | 14 | 8.98 | 8.96 | 8.87 | 8.93 | 0.06 |  |
| 14 | 6.5 | 5623 | Y | 14 | 8.97 | 8.76 | 8.74 | 8.82 | 0.13 |  |
| 14 | 6.5 | 5624 | Y | 14 | 8.99 | 8.89 | 9.10 | 8.99 | 0.11 |  |
| 14 | 6.5 | 5625 | Y | 14 | 8.98 | 8.97 | 9.32 | 9.09 | 0.20 |  |
| 22 | 6.5 | 10403S | N | 1 | 8.01 | 9.07 | 8.92 | 8.67 | 0.57 | 2.52 |
| 22 | 6.5 | 5621 | N | 1 | 7.46 | 9.10 | 9.14 | 8.57 | 0.96 | 0.58 |
| 22 | 6.5 | 5623 | N | 1 | 8.54 | 9.26 | 9.07 | 8.96 | 0.37 | 2.34 |
| 22 | 6.5 | 5624 | N | 1 | 8.77 | 9.16 | 9.22 | 9.05 | 0.25 | 3.15 |
| 22 | 6.5 | 5625 | N | 1 | 8.42 | 9.17 | 9.22 | 8.94 | 0.45 | 1.53 |
| 22 | 6.5 | 10403S | Y | 1 | 6.30 | 6.02 | 6.11 | 6.14 | 0.14 |  |
| 22 | 6.5 | 5621 | Y | 1 | 8.52 | 7.61 | 7.83 | 7.99 | 0.47 |  |
| 22 | 6.5 | 5623 | Y | 1 | 6.73 | 6.19 | 6.92 | 6.61 | 0.38 |  |
| 22 | 6.5 | 5624 | Y | 1 | 5.67 | 6.06 | 5.97 | 5.90 | 0.20 |  |
| 22 | 6.5 | 5625 | Y | 1 | 6.51 | 8.08 | 7.62 | 7.41 | 0.80 |  |
| 22 | 6.5 | 10403S | N | 7 | 8.65 | 8.91 | 7.96 | 8.50 | 0.49 | -0.31 |
| 22 | 6.5 | 5621 | N | 7 | 9.12 | 9.31 | 9.02 | 9.15 | 0.15 | 0.27 |
| 22 | 6.5 | 5623 | N | 7 | 9.07 | 9.25 | 9.01 | 9.11 | 0.13 | 0.19 |
| 22 | 6.5 | 5624 | N | 7 | 9.19 | 9.35 | 9.03 | 9.19 | 0.16 | 0.20 |
| 22 | 6.5 | 5625 | N | 7 | 9.35 | 9.08 | 8.97 | 9.13 | 0.20 | 0.22 |
| 22 | 6.5 | 10403S | Y | 7 | 8.65 | 8.87 | 8.93 | 8.82 | 0.15 |  |
| 22 | 6.5 | 5621 | Y | 7 | 8.96 | 8.90 | 8.80 | 8.89 | 0.08 |  |
| 22 | 6.5 | 5623 | Y | 7 | 8.95 | 8.88 | 8.92 | 8.92 | 0.04 |  |
| 22 | 6.5 | 5624 | Y | 7 | 9.00 | 8.99 | 8.98 | 8.99 | 0.01 |  |
| 22 | 6.5 | 5625 | Y | 7 | 8.98 | 9.02 | 8.74 | 8.91 | 0.15 |  |
| 22 | 6.5 | 10403S | N | 14 | 8.66 | 8.61 | 9.20 | 8.82 | 0.32 | -0.04 |
| 22 | 6.5 | 5621 | N | 14 | 9.77 | 9.56 | 9.39 | 9.57 | 0.19 | 0.62 |
| 22 | 6.5 | 5623 | N | 14 | 9.05 | 9.73 | 9.32 | 9.37 | 0.34 | 0.86 |
| 22 | 6.5 | 5624 | N | 14 | 9.78 | 9.41 | 9.41 | 9.53 | 0.22 | 0.47 |
| 22 | 6.5 | 5625 | N | 14 | 9.97 | 9.18 | 9.30 | 9.48 | 0.43 | 0.41 |
| 22 | 6.5 | 10403S | Y | 14 | 9.04 | 8.71 | 8.86 | 8.87 | 0.17 |  |
| 22 | 6.5 | 5621 | Y | 14 | 8.96 | 9.02 | 8.87 | 8.95 | 0.07 |  |
| 22 | 6.5 | 5623 | Y | 14 | 9.08 | 7.46 | 9.00 | 8.51 | 0.91 |  |
| 22 | 6.5 | 5624 | Y | 14 | 9.06 | 9.05 | 9.07 | 9.06 | 0.01 |  |
| 22 | 6.5 | 5625 | Y | 14 | 9.18 | 9.20 | 8.83 | 9.07 | 0.21 |  |

^1^Rep – replicates

^2^Average – calculated across each rep

^3^Std dev – standard deviation

**Supplemental Table 2**. Observed variance of *L. monocytogenes* killed by nisin extracted from cheese made at different pH

|  | Df | Sum Sq | Mean Sq | *P*-value |
| --- | --- | --- | --- | --- |
| pH | 1 | 3.79 | 3.79 | 0.31 |
| Residuals | 4 | 11.2 | 2.81 |  |

Df – degrees of freedom

Sum sq – sum of squares

Mean sq – mean squares

**Supplemental Table 3**. Observed variance of *L. monocytogenes* mutant strains killed by nisin at different pH

|  | Df | Sum Sq | Mean Sq | *P*-value |
| --- | --- | --- | --- | --- |
| Strain | 3 | 179 | 59.7 | < 0.001 |
| pH | 2 | 5.71 | 2.85 | 0.004 |
| Strain:pH | 6 | 2.44 | 0.41 | 0.443 |
| Residuals | 24 | 9.70 | 0.40 |  |

Df – degrees of freedom

Sum sq – sum of squares

Mean sq – mean squares

**Supplemental Table 4**. Observed difference within each *L. monocytogenes* mutant strain killed by nisin at different pH

| Strain | Contrast | Estimate | SE | *P*-value | Significance |
| --- | --- | --- | --- | --- | --- |
| 10403S | 5.5 – 6.0 | -0.05 | 0.52 | 0.995 |  |
|  | 5.5 – 6.5 | -0.63 | 0.52 | 0.456 |  |
|  | 6.0 – 6.5 | -0.58 | 0.52 | 0.509 |  |
| ∆*dltA* | 5.5 – 6.0 | -1.38 | 0.52 | 0.035 | * |
|  | 5.5 – 6.5 | -1.43 | 0.52 | 0.028 | * |
|  | 6.0 – 6.5 | -0.05 | 0.52 | 0.995 |  |
| ∆*mprF* | 5.5 – 6.0 | -1.34 | 0.52 | 0.041 | * |
|  | 5.5 – 6.5 | -1.07 | 0.52 | 0.121 |  |
|  | 6.0 – 6.5 | 0.28 | 0.52 | 0.854 |  |
| ∆*dltA* ∆*mprF* | 5.5 – 6.0 | -0.52 | 0.52 | 0.582 |  |
|  | 5.5 – 6.5 | -0.32 | 0.52 | 0.808 |  |
|  | 6.0 – 6.5 | 0.20 | 0.52 | 0.924 |  |

**P* < 0.05
